# *miR*-*125b*-*1* is repressed by histone modifications in breast cancer cell lines

**DOI:** 10.1186/s40064-016-2475-z

**Published:** 2016-07-02

**Authors:** Fernanda Cisneros-Soberanis, Marco A. Andonegui, Luis A. Herrera

**Affiliations:** Unidad de Investigación Biomédica en Cáncer, Instituto Nacional de Cancerología, Instituto de Investigaciones Biomédicas, Universidad Nacional Autónoma de México, Avenida San Fernando 22, Mexico City, 14080 Mexico

**Keywords:** Epigenetics, Gene promoter, Histone modifications, DNA methylation

## Abstract

**Purpose:**

Downregulation of *miR*-*125b*-*1* is associated with poor prognosis in breast cancer patients. In this work we investigated the effect of histone modifications on the regulation of this gene promoter.

**Methods and results:**

We evaluated the enrichment of two histone modifications involved in gene repression, H3K9me3 and H3K27me3, on the *miR*-*125b*-*1* promoter in two breast cancer cell lines, MCF7 (luminal A subtype) and MDA-MB-231 (triple-negative subtype), compared to the non-transformed breast cell line MCF10A. H3K27me3 and H3K9me3 were enriched in MCF7 and MDA-MB-231 cells, respectively. Next, we used an EZH2 inhibitor to examine the reactivation of *miR*-*125b*-*1* in MCF7 cells and evaluated the transcriptional levels of pri-miR-125b-1 and mature miR-125b by qRT-PCR. pri-miRNA and mature miRNA transcripts were both increased after treatment of MCF7 cells with the EZH2 inhibitor, whereas no effect on *miR*-*125b*-*1* expression levels was observed in MDA-MB-231 and MCF10A cells. We subsequently evaluated the effect of *miR*-*125b*-*1* reactivation on the expression and protein levels of BAK1, a target of miR-125b. We observed 60 and 70 % decreases in the expression and protein levels of BAK1, respectively, compared to cells that were not treated with the EZH2 inhibitor. We over-expressed KDM4B/JMJD2B to reactivate this miRNA, resulting in a three-fold increase in miR-125b expression compared with the same cell line without KDM4B/JMJD2B over-expression.

**Conclusion:**

The *miR*-*125b*-*1* is repressed by different epigenetic mechanisms depending on the breast cancer subtype and that *miR*-*125b*-*1* reactivation specifically eliminates the effect of repressive histone modifications on the expression of an pro-apoptotic target.

**Electronic supplementary material:**

The online version of this article (doi:10.1186/s40064-016-2475-z) contains supplementary material, which is available to authorized users.

## Background

MicroRNAs (miRNAs) are short single-stranded RNAs that could regulate gene expression at the post-transcriptional level. miRNAs are typically transcribed as primary transcripts (pri-miRNAs) that are subsequently matured in a multi-step biogenesis process to generate the functional form or mature miRNA (Lee and Dutta 2009). Accumulating evidence indicates that *miRNA* deregulation is related to pathologies including cancer. Some miRNAs are associated with tumor suppressor or oncogene activity, depending on the downregulated target (Di Leva et al. 2014).

In particular, miR-125b-1 exhibits tumor suppressor activity in some types of cancer, including breast, ovarian and bladder cancer (Banzhaf-Strathmann 2014). *mir*-*125b*-*1* may be involved in biological processes such as apoptosis, cell proliferation and cell migration because it regulates genes such as *BAK1* (Zhou et al. 2010), *ERBB2* (Scott et al. 2007) and *ETS1* (Zhang et al. 2011), respectively. In most breast cancer tumors, *miR*-*125b*-*1* downregulation is associated with a poor prognosis. *miR*-*125b*-*1* can be repressed by DNA methylation in the promoter region (Zhang et al. 2011; Soto-Reyes et al. 2012). However, the *miR*-*125b*-*1* promoter is within a CpG island promoter with intermediate CpG content. Promoters with intermediate CpG content are typically regulated by DNA methylation and histone modifications (Weber et al. 2007). Thus, we were interested in evaluating the relevance of repressive histone modifications in *miR*-*125b*-*1* downregulation.

In this study, we evaluated repressive histone modifications in the promoter regions of *miR*-*125b*-*1* and the effect on the transcriptional regulation of this gene. Chromatin immunoprecipitation revealed H3K9me3 and H3K27me3 histone modifications associated with gene repression in the *miR*-*125b*-*1* promoter in breast cancer cell lines. These histone modifications were then removed by over-expressing KDM4B/JMJD2B to remove H3K9me3 (Fodor et al. 2006) or using an EZH2 inhibitor to remove H3K27me3 (McCabe et al. 2012). Finally, we evaluated *miR*-*125b*-*1* reactivation by quantitative real-time PCR (qRT-PCR) and the effect on a target of this miRNA.

## Methods

### Human breast cell lines and treatment

The human breast cancer cell lines MCF7 and MDA-MB-231 were cultured in DMEM/F12 supplemented with 10 % fetal bovine serum (GIBCO). MCF10A, a non-transformed cell line, was cultured in DMEM/F12 (3:1) supplemented with 10 % fetal bovine serum (GIBCO), 2 mM glutamine (GIBCO), 10 ng/mL EGFrh (Invitrogen), 120 mU/mL human recombinant insulin (Insulinex) and 1 µg/mL hydrocortisone (SIGMA).

To eliminate the H3K9me3 histone modification, MCF10A and MDA-MB-231 were transfected with a plasmid encoding KDM4B/JMJD2B-GFP (Fodor et al. 2006) using Lipofectamine LTX (Invitrogen). As a control, the same cell lines were transfected with empty plasmid. Plasmid-carrying cells were selected by cell sorting using a FACSAria III cytometer (BD). To eliminate the H3K27me3 histone modification, the breast cell lines were incubated with 200, 500, 1000 or 2000 nM GSK126 for 4 days.

### RNA isolation and qRT-PCR expression analysis

Total RNA was isolated from cells using TRIzol reagent (Invitrogen). pri-miR-125b-1 and pri-miR-125b-2 expression levels were evaluated by qRT-PCR using SYBR Green (ThermoFisher Scientific). Mature *miR*-*125b* expression levels were quantified by qRT-PCR using a TaqMan assay according to the manufacturer’s protocol (Applied Biosystems). One hundred nanograms of total RNA were reverse transcribed using specific stem-loop RT primers. Next, the products were amplified and detected by PCR with specific primers and TaqMan probes (*miR*-*125b*, Applied Biosystems). *U6* snRNA served as an internal normalized reference. The amplification and detection of specific products were calculated by the 2^−ΔΔCt^ method.

### Western blot analysis

Cells were grown in 10-cm^2^ dishes and lysed using Cell Lysis Buffer (Cell Signaling) with protease inhibitor cocktail (Cell Signaling) and 100 µM PMSF (SIGMA). Protein samples were resolved on 12 % Tris–glycine gels and transferred to PVDF membranes. The membranes were then incubated with α-H3K27me3 (1:1000; Millipore, 07-449), α-H3 (1:1000; SIGMA, H0164), α-BAK1 (Santa Cruz, sc-832) and α-GAPDH (Santa Cruz, sc-25778). After washing with TBS, the membranes were incubated with peroxidase-conjugated goat anti-rabbit antibody (1:20,000; Novus, NB7187) followed by chemiluminescence staining (Millipore).

### Chromatin immunoprecipitation assay

The chromatin immunoprecipitation (ChIP) assay was performing using the OneDay ChIP kit (Diagenode) according to the manufacturer’s protocol. To immunoprecipitate H3K9me3 and H3K27me3, we used 4 µg each of α-H3K9me3 (Abcam, ab-8898) and -H3K27me3 (Millipore, 07-449), respectively. The immunoprecipitated DNA was analyzed by PCR using primers specific for the *miR*-*125b1* promoters. As positive ChIP controls, we used SOX-9 and MYT-1 promoters.

### Statistical analysis

Data from at least three independent experiments are expressed as the mean ± standard deviation. Differences between groups were analyzed using Student’s *t* test. Data were considered significant at p < 0.05.

## Results

### *miR*-*125b*-*1* is downregulated in breast cancer cell lines

**Fig. 1:**
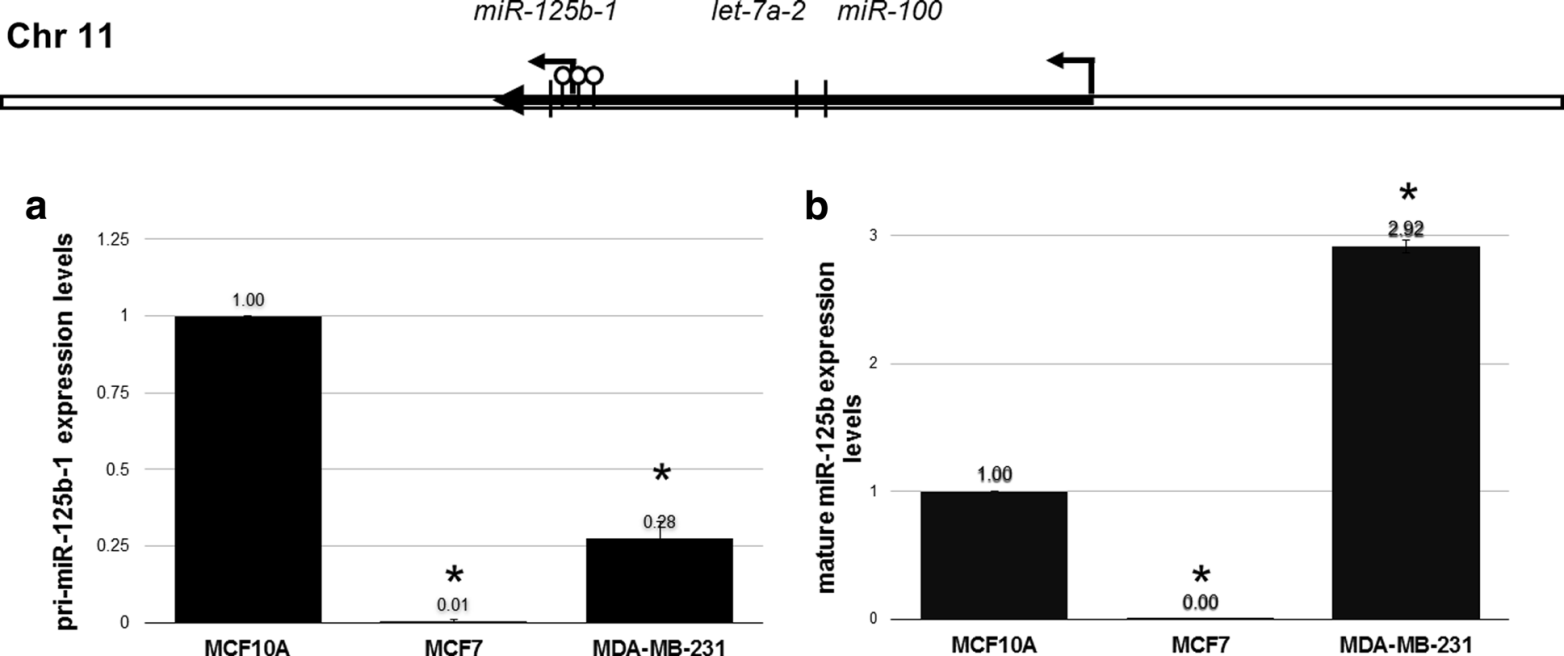
The miR-125b-1 precursor and mature transcript are downregulated in the MCF7 breast cancer cell line. We evaluated the expression levels of pri-miR-125b-1 and mature miR-125b in the MCF10A non-transformed breast cell line and the two breast cancer cell lines MCF7 and MDA-MB-231. *p > 0.001

miR-125b (mature miRNA) is transcribed from two different genes: *miR*-*125b*-*1* (chromosome 11) and *miR*-*125b*-*2* (chromosome 21). However, the transcriptional activity of *miR*-*125b*-*2* is low (Additional file 1: Fig. 1). Thus, most miR-125b is derived from the *miR*-*125b*-*1* gene. To determine *miR*-*125b*-*1* expression levels, we evaluated pri-miRNA and mature miRNA levels in the breast cancer cell lines MCF7 and MDA-MB-231 by qRT-PCR compared with the non-transformed breast cell line MCF10A. pri-miR-125b-1 levels in MCF7 and MDA-MB-231 cells were reduced by 99 and 72 %, respectively, compared with MCF10A cells. However, mature miR-125b levels were reduced only in MCF7 cells. In MDA-MB-231 cells, mature miR-125b levels were increased by nearly threefold (Fig. 1). This increment of miR-125b in MDA-MB-231 cells may be associated with the accumulation of miRNA transcripts.

### Histone modification marks in *miR*-*125b*-*1* promoter regions

**Fig. 2:**
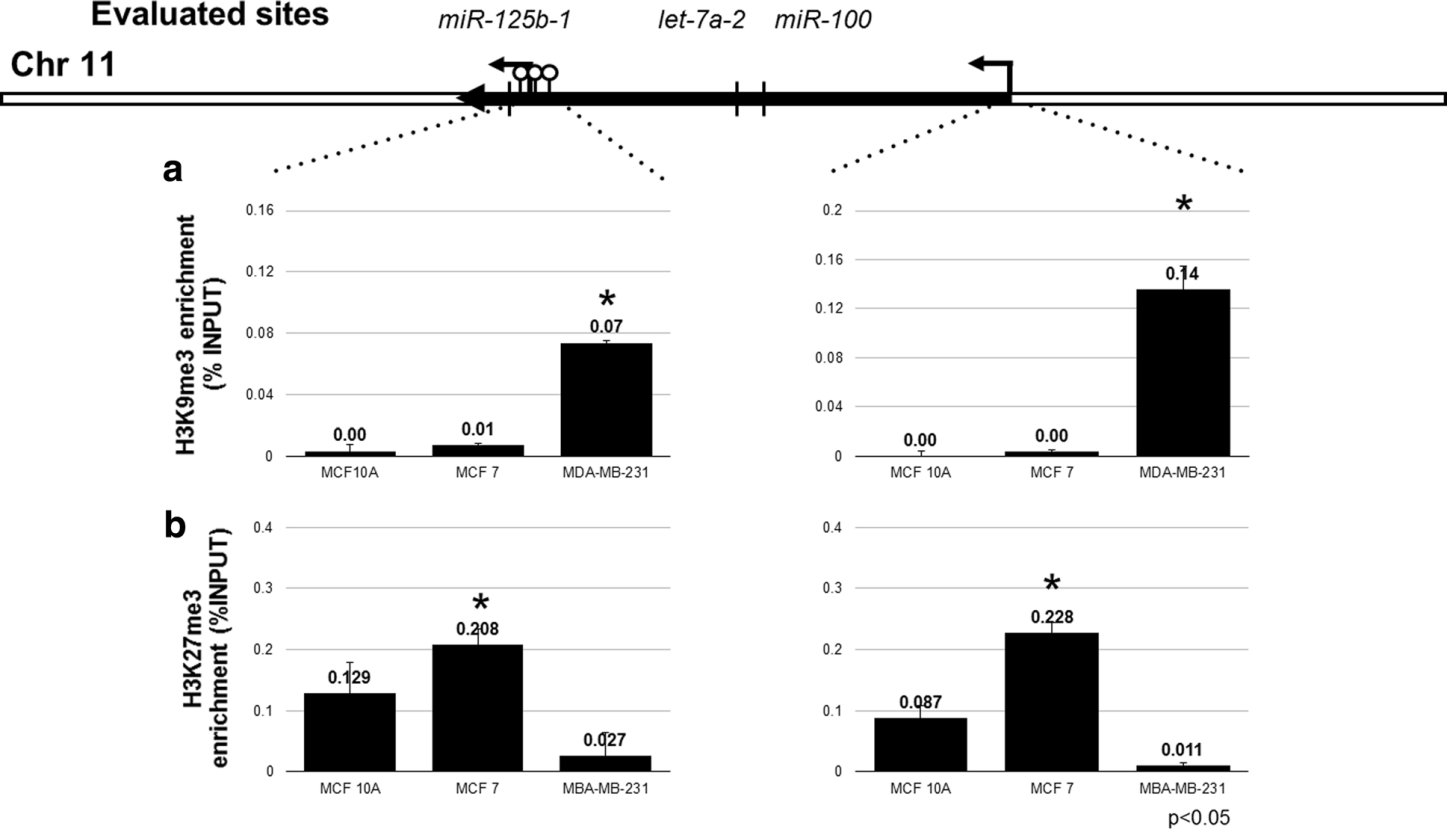
H3K9me3 and H3K27me3 are enriched on miR-125b-1 promoters in MDA-MB-231 and MCF7 breast cancer cells, respectively. To determine which histone modification was involved in miR-125b-1 repression, we evaluated H3K9me3 (**a**) and H3K27me3 (**b**) expression in the promoter regions by chromatin immunoprecipitation

Two promoters are associated with *miR*-*125b*-*1* transcriptional regulation. The first is in a CpG island close to the *miR*-*125b*-*1* sequence (Wang et al. 2013). This CpG island has an intermediate CpG content, and thus the transcriptional regulation of *miR*-*125b*-*1* may be associated with DNA methylation and histone modifications (Weber et al. 2007; Marson et al. 2008). The second promoter is 55 kb upstream from the *miR*-*125b*-*1* sequence. This promoter, which may regulate the transcription of the miR-125b-1, let-7a-2 and miR-100 genes (Chien et al. 2011), is not in a CpG island, and thus the regulation of these genes may be associated with histone modifications (Fig. 2).

To further investigate the repressive mechanism of *miR*-*125b*-*1* in breast cancer cell lines, we evaluated two histone modifications involved in gene repression: H3K9me3 and H3K27me3. H3K9me3 and H3K27me3 are associated with constitutive and facultative heterochromatin, respectively. We observed enrichment of H3K9me3 in both promoters in MDA-MB-231 cells but not MCF7 and MCF10A cells (Fig. 2a). However, in MCF7 cells, we observed twofold enrichment of H3K27me3 compared with MCF10A cells. We did not observe this enrichment on *miR*-*125b*-*1* promoters in MDA-MB-231 cells (Additional file 2: Fig. 2; Fig. 2b).

### *miR*-*125b*-*1* reactivation in MDA-MB-231 by KDM4B/JMJD2B over-expression

**Fig. 3:**
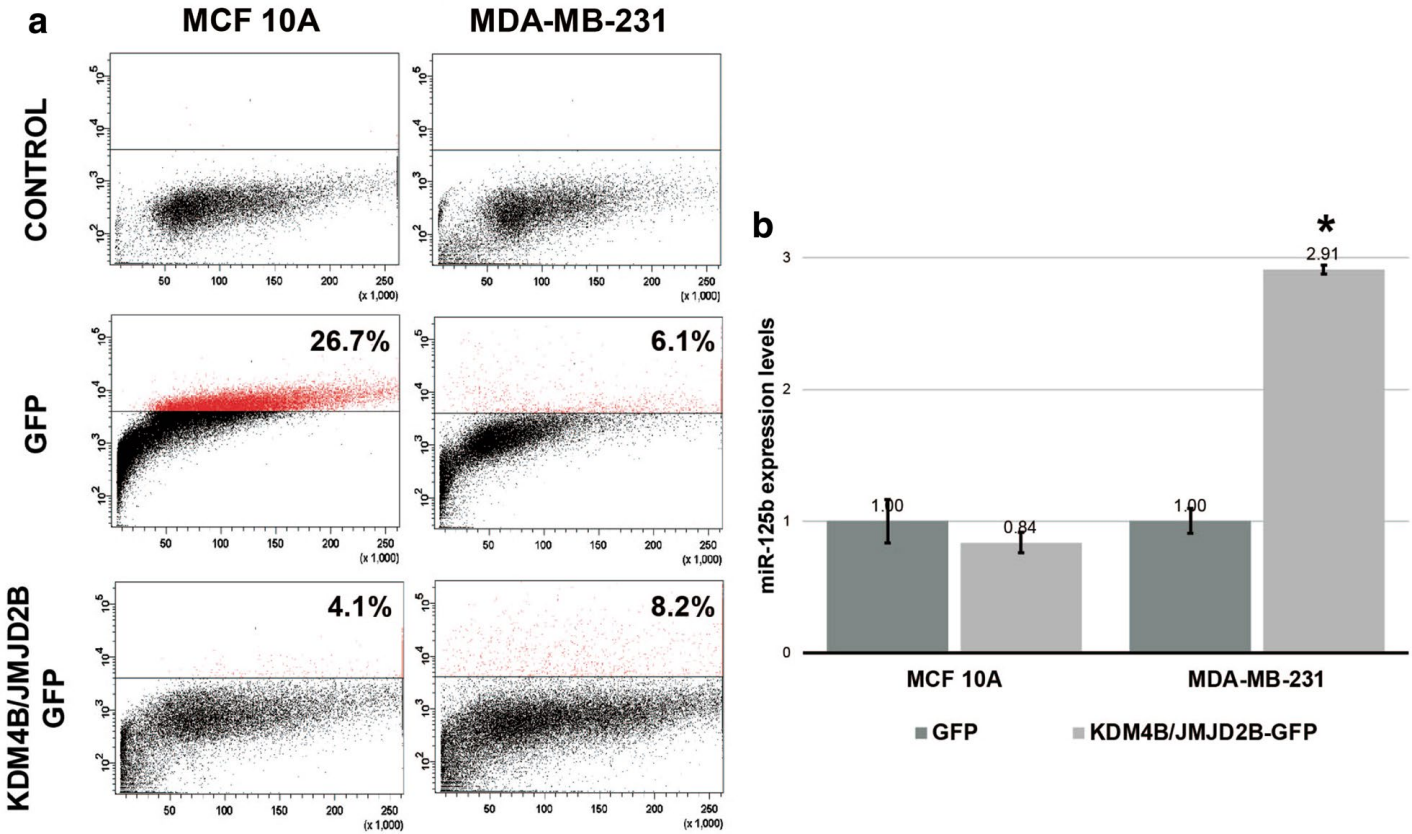
JMJD2B over-expression reactivates miR-125b in MDA-MB-231 breast cancer cells. To determine if H3K9me3 is involved in miR-125b-1 repression, we over-expressed JMJD2B in MCF10A and MDA-MB-231 breast cells and then selected cells over-expressing JMJD2B by sorting (**a**). After the selection, we evaluated the expression levels of miR-125b (**b**)

To determine the role of H3K9me3 in MDA-MB-231 cells, we over-expressed KDM4B/JMJD2B to reduce global levels of H3K9me3 histone modification in MCF10A and MDA-MB-231 cells. We specifically selected transfected cells over-expressing KDM4B/JMJD2B by cell sorting (Fig. 3a). Subsequent qRT-PCR analysis revealed a three-fold increase in miR-125b levels in KDM4B/JMJD2B-transfected MDA-MB-231 cells compared with MDA-MB-231 cells transfected with empty vector. However, no significant differences in miR-125b levels were observed between KDM4B/JMJD2B-transfected MCF10A cells and empty vector-transfected MDA-MB-231 cells (Fig. 3b).

### *miR*-*125b*-*1* is reactivated by an EZH2 inhibitor in MCF7 cells

**Fig. 4:**
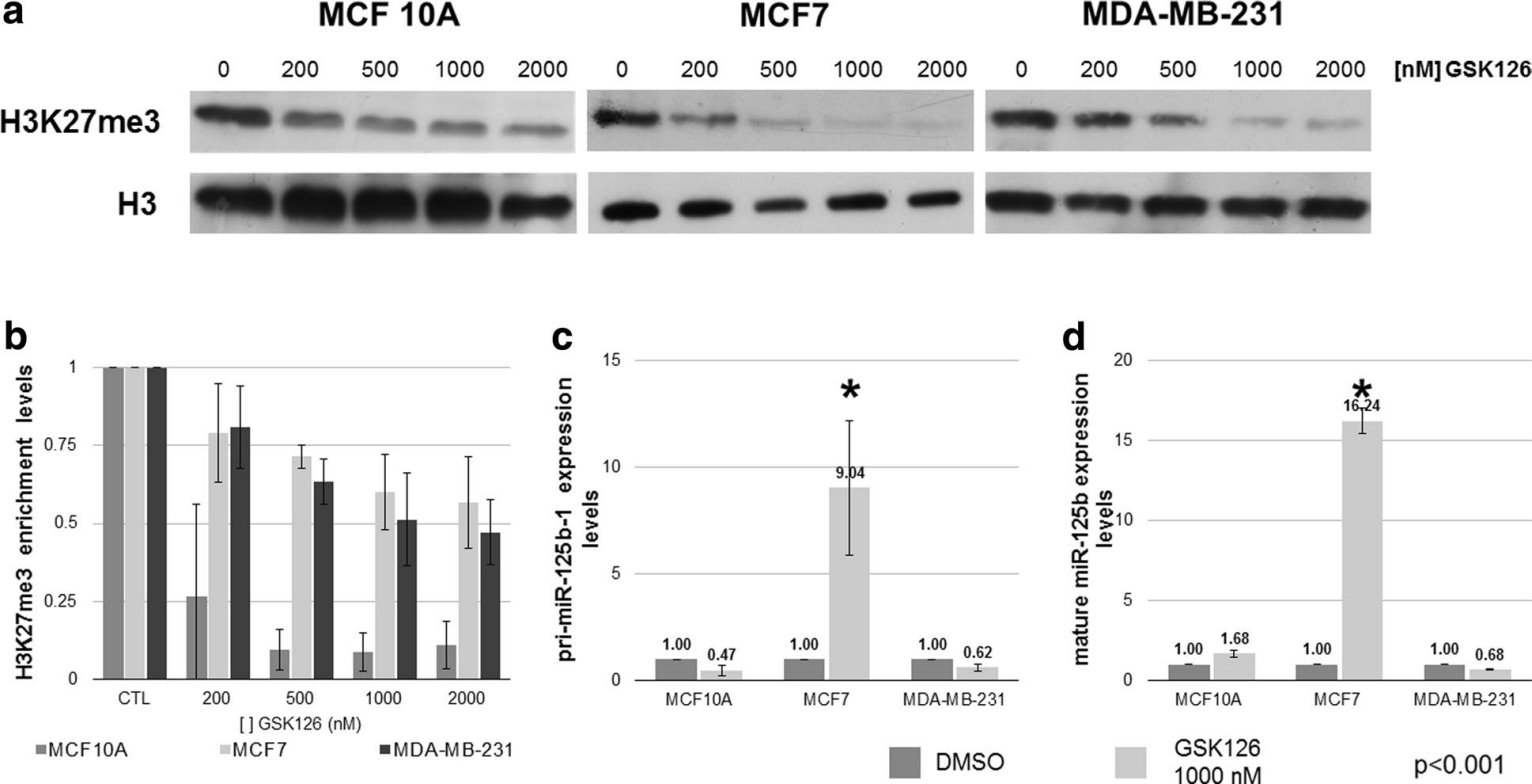
An inhibitor of EZH2 reactivates miR-125b-1 in MCF7 breast cancer cells. To determine the dose of GSK126 that reduces H3K27me3 levels, we treated the breast cell lines with different GSK126 concentrations and performed Western blot analysis (**a**). **b** Densitometric analysis of triplicate Western blots. We chose the 1000-nM dose to evaluate pri-miR-125b-1 (**c**) and mature miR-125b expression levels (**d**)

To determine if H3K27me3 is responsible for *miR*-*125b*-*1* repression, we treated the breast cell lines with the EZH2 inhibitor GSK126. GSK126 is more than 1000-fold selective for EZH2 versus 20 other human methyl transferases (McCabe et al. 2012). To choose an effective GSK126 concentration without altering other methyltransferases, we constructed a dose curve to determine the minimum GSK126 concentration at which global H3K27me3 was decreased. We estimated global H3K27me3 levels by Western blotting and determined that 1000 nM was the minimum effective GSK126 concentration to evaluate *miR*-*125b*-*1* reactivation in breast cancer cell lines (Fig. 4a, b). Subsequent qRT-PCR analysis revealed that the expression levels of pri-miR-125b-1 (primary transcript) and miR-125b (mature transcript) were increased ninefold (Fig. 4c) and 16-fold (Fig. 4d), respectively, in MCF7 cells compared untreated MCF7 cells. Interestingly, no changes in pri-miR-125b-1 (Fig. 4c) and mature miR-125b (Fig. 4d) transcription levels were observed in MDA-MD-231 and MCF10A cells. Hence, we conclude that H3K27me3 is responsible for *miR*-*125b*-*1* repression in MCF7 cells.

### *miR*-*125b*-*1* reactivation affects the expression levels of BAK1

**Fig. 5:**
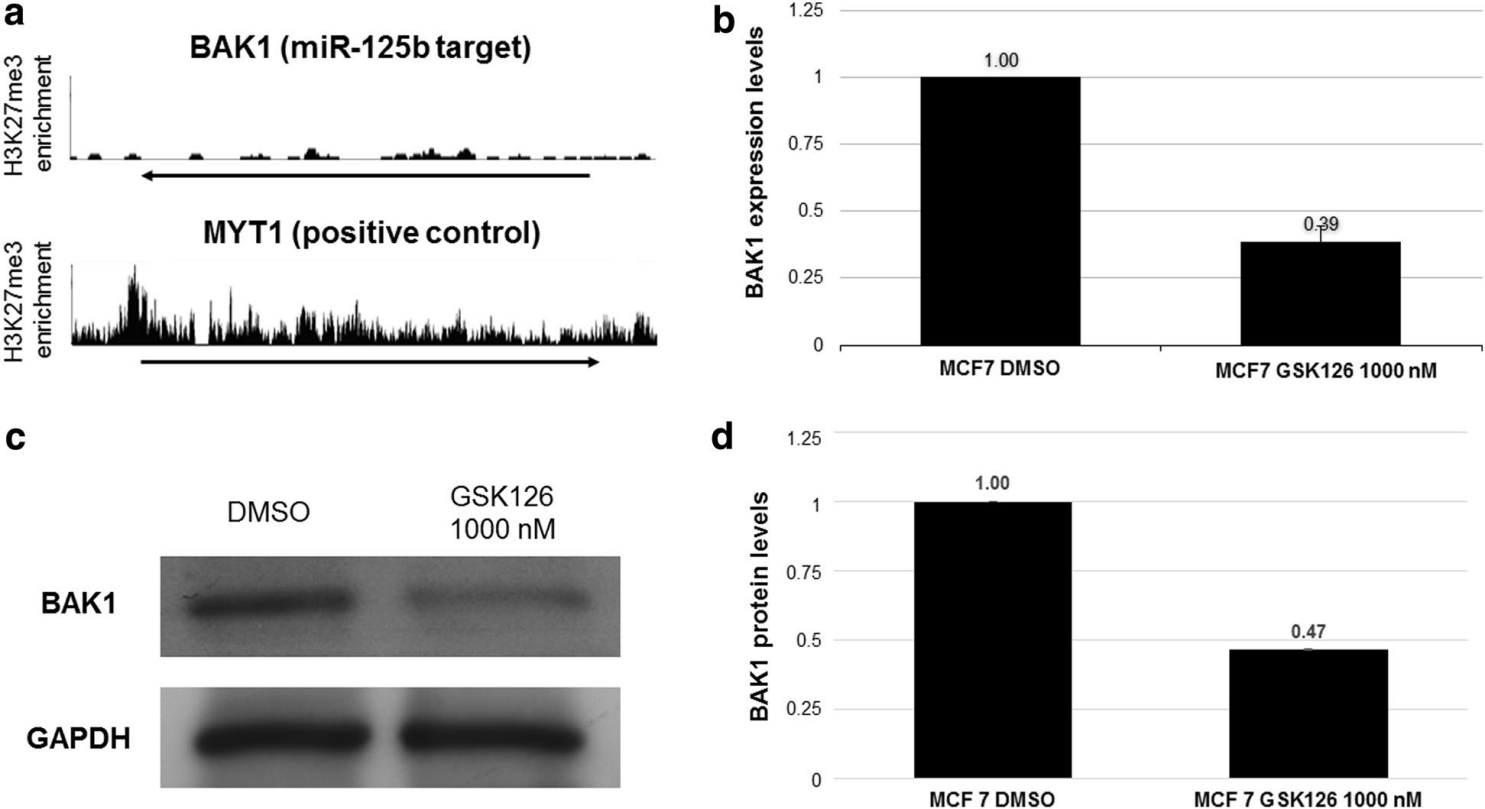
Elevated levels of miR-125b-1 affect the expression levels of BAK1. The target BAK1 was selected based on an evaluation of H3K27me3 enrichment in MCF7 cells by ENCODE (**a**). Next, we evaluated BAK1 expression levels by qRT-PCR (**b**). Finally, we determined protein levels by Western blotting (**c**, **d**). n = 3 *p > 0.05

*mir*-*125b* is involved in biological processes such as apoptosis, cell proliferation and cell migration-regulating genes such as *BAK1* (Zhou et al. 2010), *ERBB2* (Scott et al. 2007) and *ETS1* (Zhang et al. 2011), respectively. We therefore sought to evaluate the effects of *miR*-*125b*-*1* reactivation on the expression and protein levels of its targets. Using the ENCODE database, we evaluated the presence of H3K27me3 on some miR-125b target gene promoters in the MCF7 cell line (Additional file 3: Fig. 3). This histone modification was absent only in *BAK1* (Fig. 5a). This analysis is important because GSK126 treatment of MCF7 cells may alter the expression and protein levels of target genes via an miR-125b-independent mechanism.

We therefore evaluated BAK1 transcription levels in MCF7 cells treated with and without GSK126 by qRT-PCR. We observed a 71 % reduction in BAK1 expression levels in MCF7 cells treated with GSK126 (Fig. 5b). Next, we determined the proteins levels of BAK1 by Western blotting. We observed a 53 % reduction in BAK1 protein levels in MCF7 cells treated with GSK126 (Fig. 5c, d). We therefore conclude that *miR*-*125b*-*1* reactivation affects the expression and protein levels of BAK1, an miR-125b target.

## Discussion

miR-125b is an miRNA that may be involved indirectly in biological processes such as apoptosis, cell proliferation and cell migration (Banzhaf-Strathmann 2014). *miR*-*125b*-*1* downregulation has been associated with increased cell proliferation (Scott et al. 2007), metastasis (Zhang et al. 2011) and drug resistance (Zhou et al. 2010; Wang et al. 2013; Vilquin et al. 2015) in breast cancer. *miR*-*125b*-*1* repression has been associated with DNA methylation on its promoter region (Zhang et al. 2011; Soto-Reyes et al. 2012). However, in this study, we were interested in evaluating the relationship between *miR*-*125b*-*1* repression and repressive histone modifications such as H3K9me3 and H3K27me3. As a study model, we used two breast cancer cell lines, MCF7 (luminal A subtype) and MDA-MB-231 (triple-negative subtype), and the non-transformed breast cell line MCF10A.

We detected H3K9me3 and H3K27me3 in the *miR*-*125b*-*1* promoter region in MDA-MB-231 and MCF7 cells, respectively. These histone modifications were mutually exclusive and did not coexist. These repressive histone modifications are related to different silencing mechanisms. The H3K9me3 repressive histone modification is associated with constitutive heterochromatin. Histone methyltransferases, such as G9a and SUV39H1, are involved in “writing this mark”. H3K9me3 is commonly localized in repeat regions but is not usually observed in gene-rich regions (Kim 2012). Thus, the presence of H3K9me3 on the *miR*-*125b*-*1* promoter in MDA-MB-231 cells is unusual. However, the presence of this histone modification is important in *miR*-*125b*-*1* transcriptional regulation because when removal of the H3K9me3 mark reactivated this miRNA (Fig. 3).

By contrast, the H3K27me3 repressive histone modification is associated with facultative heterochromatin and the presence of the Polycomb complex. This repressive histone modification is generally localized in gene-rich regions (Kim 2012; Chase and Cross 2011). In breast cancer tumors, H3K27me3 has been related to clinical stage and estrogen-receptor positive tumors (Healey et al. 2014). Some groups have reported an increase in the transcriptional levels of EZH2 (a H3K27 histone methyl-transferase) in the luminal breast cancer subtype (Healey et al. 2014), and an association between increased EZH2 activity and poor prognosis has been reported (Jene-Sanz et al. 2013; Holm et al. 2012). These analyses are relevant to our work because *miR*-*125b*-*1* is an EZH2 target in the luminal breast cancer cell line MCF7. *miR*-*125b*-*1* repression by EZH2 may be involved in drug resistance because miR-125b can downregulate *BAK1*, an anti-apoptosis gene (Zhou et al. 2010). We demonstrated that the reactivation of *miR*-*125b*-*1* decreases BAK1 expression and protein levels (Figs. 4, 5). This result suggests that a decrease in *BAK1* may increase the sensitivity of cells to Taxol treatment (Zhou et al. 2010).

We conclude that *miR*-*125b*-*1* is transcriptionally regulated by histone modifications such as H3K9me3 and H3K27me3, depending on the breast cancer cell subtype. In the luminal breast cancer cell line, we demonstrated that the reactivation of this miRNA affects the expression and protein levels of *BAK1*, a target involved in anti-apoptosis.

## Additional files

10.1186/s40064-016-2475-z pri-miR-125b-1 and pri-miR-125b-2 expression levels in breast cancer cell lines. We evaluated pri-miR-125b1 and pri-miR-125b-2 transcriptional levels by qRT-PCR in MCF10A, a non-transformed breast cell line, and MCF7 and MDA-MB-231, two breast cancer cell lines. *p>0.001.
10.1186/s40064-016-2475-z Positive controls for H3K9me3 and H3K27me3 chromatin immunoprecipitation.
10.1186/s40064-016-2475-z H3K27me3 enrichment in miR-125b gene target promoters. We used the ENCODE database to evaluate the presence of H3K27me3 in miR.
