# Supplementary figures and images for: miR-125b-1 is repressed by histone modifications in breast cancer cell lines
Source: Springerplus. 2016 Jul 2;5(1):959. doi: 10.1186/s40064-016-2475-z (PMC4930440; doi:10.1186/s40064-016-2475-z)

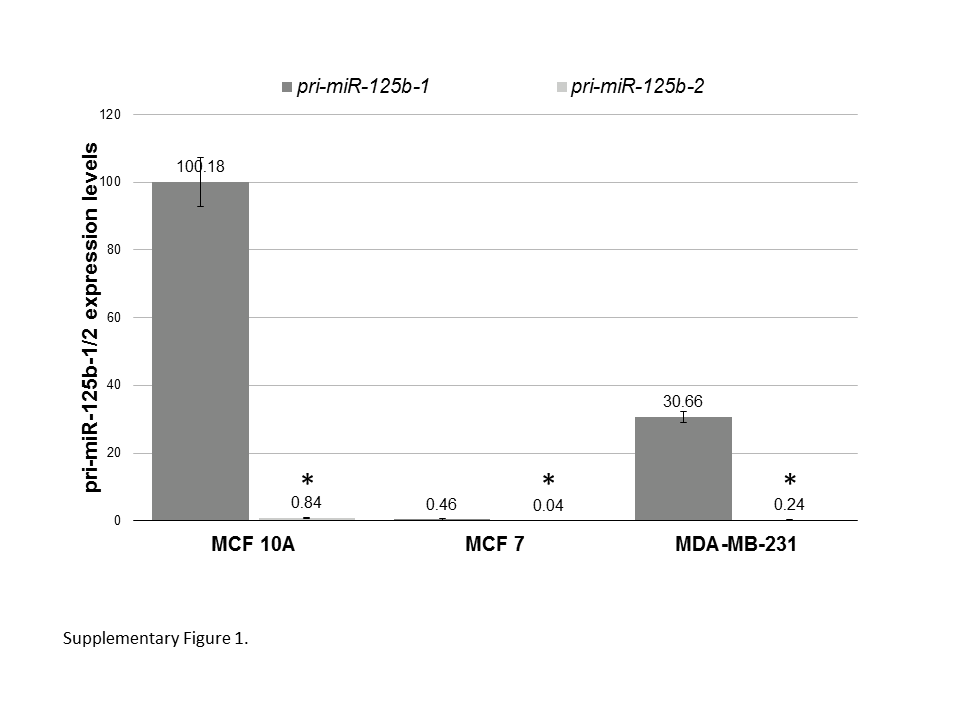

Supplement: Supplementary file 1 — 10.1186/s40064-016-2475-z pri-miR-125b-1 and pri-miR-125b-2 expression levels in breast cancer cell lines. We evaluated pri-miR-125b1 and pri-miR-125b-2 transcriptional levels by qRT-PCR in MCF10A, a non-transformed breast cell line, and MCF7 and MDA-MB-231, two breast cancer cell lines. *p>0.001. [file 40064_2016_2475_MOESM1_ESM.tif]

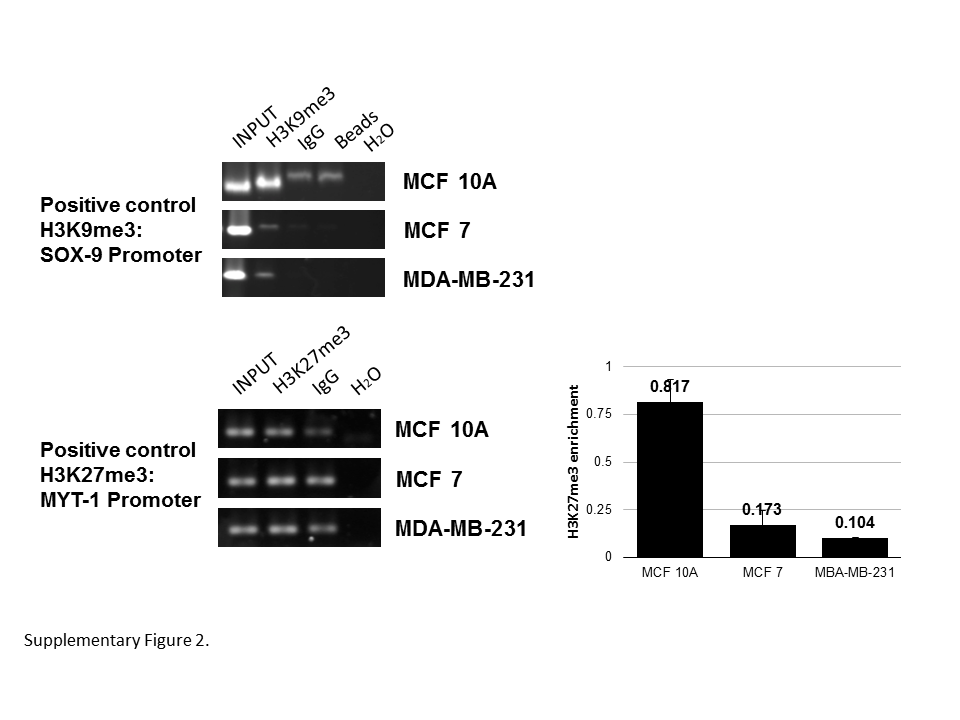

Supplement: Supplementary file 2 — 10.1186/s40064-016-2475-z Positive controls for H3K9me3 and H3K27me3 chromatin immunoprecipitation. [file 40064_2016_2475_MOESM2_ESM.tif]

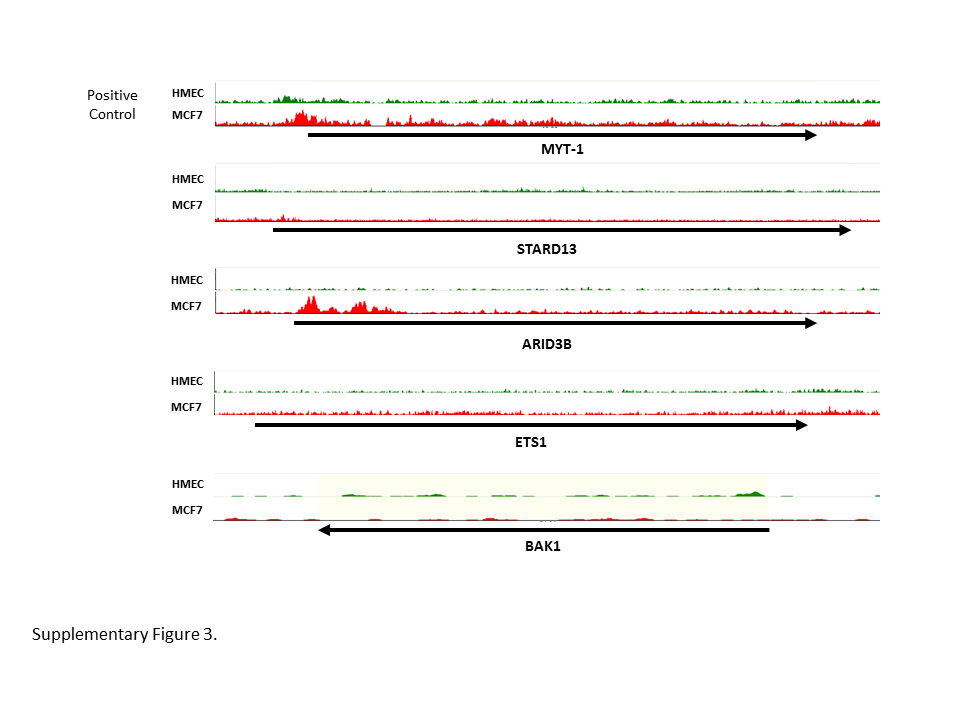

Supplement: Supplementary file 3 — 10.1186/s40064-016-2475-z H3K27me3 enrichment in miR-125b gene target promoters. We used the ENCODE database to evaluate the presence of H3K27me3 in miR. [file 40064_2016_2475_MOESM3_ESM.tif]
